# Effect of Bacteriophage Infection in Combination with Tobramycin on the Emergence of Resistance in *Escherichia coli* and *Pseudomonas aeruginosa* Biofilms

**DOI:** 10.3390/v6103778

**Published:** 2014-10-03

**Authors:** Lindsey B. Coulter, Robert J. C. McLean, Rodney E. Rohde, Gary M. Aron

**Affiliations:** 1 Clinical Laboratory Science Program, Texas State University, 601 University Drive, San Marcos, TX 78666, USA; E-Mails: lcoulter87@gmail.com (L.B.C.); rrohde@txstate.edu (R.E.R.); 2 Department of Biology, Texas State University, San Marcos, 601 University Drive, TX 78666, USA; E-Mail: McLean@txstate.edu (R.J.C.M.)

**Keywords:** biofilms, bacteriophage, antibiotic, resistance, mixed therapy

## Abstract

Bacteriophage infection and antibiotics used individually to reduce biofilm mass often result in the emergence of significant levels of phage and antibiotic resistant cells. In contrast, combination therapy in *Escherichia coli* biofilms employing T4 phage and tobramycin resulted in greater than 99% and 39% reduction in antibiotic and phage resistant cells, respectively. In *P. aeruginosa* biofilms, combination therapy resulted in a 60% and 99% reduction in antibiotic and PB-1 phage resistant cells, respectively. Although the combined treatment resulted in greater reduction of *E. coli* CFUs compared to the use of antibiotic alone, infection of *P. aeruginosa* biofilms with PB-1 in the presence of tobramycin was only as effective in the reduction of CFUs as the use of antibiotic alone. The study demonstrated phage infection in combination with tobramycin can significantly reduce the emergence of antibiotic and phage resistant cells in both *E. coli* and *P. aeruginosa* biofilms, however, a reduction in biomass was dependent on the phage-host system.

## 1. Introduction

Bacterial biofilms are populations of cells adherent to an abiotic or biotic surface that can grow to be several millimeters thick [1]. Biofilms, such as those formed by *Escherichia coli* and *Pseudomonas aeruginosa*, have been found on medical implants, such as catheters and artificial hips [2,3], as well as pulmonary infections within lungs of cystic fibrosis patients [2,4,5]. The community of cells are coated in a sticky matrix made of extracellular DNA, secreted proteins, and polysaccharides [4,6,7] collectively called extracellular polymeric substances (EPS) that allow for the adherence to surfaces as well as provide protection from antimicrobial agents [8]. Biofilms have been shown to be highly tolerant to antimicrobials [4,9], a feature that was first ascribed to the ability of the biofilm matrix to restrict antimicrobial penetration [10]. Other mechanisms that have been described include the establishment of slow-growing antibiotic-resistant subpopulations within biofilms (persister cells) [11], and biofilm-specific gene expression [12].

Infections due to biofilms are difficult to treat because of their high tolerance to antimicrobials [13]. Bacteriophage therapy has been suggested for the treatment of biofilms [14]. Although effective in decreasing biofilm mass, phage therapy alone has not been shown to eradicate biofilms [14,15]. Phage therapy was a common method of treating infections before replaced by the discovery of antibiotics, and is still being used in parts of Eastern Europe for the treatment of a variety of afflictions ranging from acne and urinary tract infections to methicillin-resistant *Staphylococcus aureus* [14,15]. A major concern of phage therapy is the development of phage resistance. To reduce the chance of resistant cells from emerging, combination therapy is often used which is more effective than the use of a single treatment [16,17]. Tré-Hardy *et al*. [16] demonstrated the use of tobramycin in combination with clarithromycin to be more effective in decreasing mature *P. aeruginosa* biofilms and Parra-Ruiz *et al*., [18] found combinations of daptomycin or moxifloxacin with clarithromycin to be effective at decreasing *Staphylococcus aureus* biofilms. In phage therapy, Fu *et al*. [19] found pre-treating catheters with a cocktail of bacteriophage can decrease the formation of *P. aeruginosa* biofilms compared to the use of a single phage. There have also been reports on phage and antibiotic combinational therapy on biofilms. Bedi *et al*. [20] found amoxicillin and lytic phage on *Klebsiella pneumoniae* B5055 biofilms produced a greater reduction in biofilm (~1–2 log) compare to either treatment alone. Verma *et al*. [21] continued the work on *K. pneumoniae* B5055 using ciprofloxacin and depolymerase-producing phage KPO1K2 and found the combination to be significantly more effective. *S. aureus* biofilms treated with phage SAP-26 and rifampicin reduced the biofilm by ~5 logs, while phage SAP-26 and rifampicin only reduced the biofilm by ~3 log and ~4 log, respectively [22]. There are no reports on the emergence of resistance in biofilms treated with a combination of phage and antibiotics. This study compares the effect of bacteriophage infection with tobramycin on the emergence of phage and antibiotic resistant cells and biofilm survival. The results demonstrated in both *E. coli* and *P. aeruginosa* biofilms phage infection in combination with tobramycin reduced the emergence of antibiotic and phage resistant cells, however, biomass reduction was dependent on the phage-host system.

## 2. Materials and Methods

### 2.1. Bacteria and Bacteriophage

*Escherichia coli* B (ATCC 11303) and *Pseudomonas aeruginosa* PAO1 (obtained from V. Deretic, University of New Mexico) were grown in Luria-Bertani (LB) broth (Accumedia Manufacturers, Inc., Lansing, Michigan, MI, USA) at 37 °C in an orbital rotating shaker water bath (Lab-Line Instruments, Inc. model 3540 Orbital Shaker Bath, Melrose Park, IL, USA). Bacteriophage T4 (ATCC 11303-B4) was used to infect *E. coli* and bacteriophage PB-1 [23] (ATCC 15692-B3) was used to infect *P. aeruginosa*. Bacteriophage T4 and PB-1 stocks were prepared by infecting early log phase of *E. coli* or *P. aeruginosa*, respectively, at a multiplicity of infection (MOI) of approximately 1000. Infected cultures were placed into a 37 °C reciprocal shaking water bath (Blue M Electric Company Magni Whirl MSB-1122A-1 Shaker Bath, Blue Island, IL, USA) until the cultures cleared, or 2 h. Infected cultures were then placed into a glass centrifuge tube with 0.5 mL of chloroform at 4 °C. After 5 min at 4 °C the infected cultures were shaken for 1 min and placed at 4 °C for 5 min. The cultures were centrifuged (Eppendorf Centrifuge model 5810 R, Hamburg, Germany) at 3000 × *g* for 20 min at 4 °C. The supernatant was filtered (0.45 µm) and phage titers were determined by a soft-agar overlay plaque assay [24].

### 2.2. Antibiotic Preparation

Tobramycin (T4014, Sigma-Aldrich Co., St. Louis, MO, USA) stock solution of 10 mg.mL^−1^ were prepared by diluting tobramycin in deionzed water and filter sterilizing (0.22 µm; Fisher 25 mm syringe filter; ThermoFisher Scientific Inc., Waltham, MA, USA).

### 2.3. Biofilm Growth

Silicone rubber disks, 7 × 1 mm (Dapro Rubber Inc., Tulsa, OK, USA), were placed into 50 mL of LB broth and inoculated with overnight cultures of *E. coli* or *P. aeruginosa* to a cell density 10^6^ CFU.mL^−1^ (OD_600nm_ = 0.1). Monocultures were incubated in an orbital shaking water bath at 100 rpm for 48 h at 37 °C. Biofilm growth was measured by removing colonized disks from the culture vessel, dipping in sterile phosphate buffered saline (PBS) (Sigma-Aldrich, Co., St Louis, MO, USA) to remove unattached bacteria, then placing in a scintillation vial containing 5 mL H_2_O and using the bath sonication, dilution plating protocol described by Corbin *et al*. [25].

### 2.4. Determination of MOI and Antibiotic Concentration on Cell Survival

*E. coli* and *P. aeruginosa* biofilms were grown as described previously. After 48 h, disks containing biofilms were rinsed in phosphate buffered saline (PBS) (Sigma-Aldrich, Co., St Louis, MO, USA) and placed in individual 20 mL scintillation vials containing LB broth and phage at various MOI (0.0001–10) or tobramycin (0.25–4 µg.mL^−1^) [26]. Treated biofilms were incubated in a reciprocal shaking water bath at 100 rpm for 24 h at 37 °C. Biofilm enumeration following treatments were performed as described in Section 2.3. Cell density was determined by dilution plating on LB agar plates.

### 2.5. Antibiotic Treatment

Forty-eight-hour biofilms were gently rinsed with 5 mL of PBS in sterile test tubes to remove planktonic cells. Biofilms were placed in individual 20 mL scintillation vials containing 10 mL of LB broth with 2 µg.mL^−1^ of tobramycin for *E.* coli and 0.5 µg.mL^−1^ of tobramycin for *P. aeruginosa*. Biofilms were incubated at 37 °C at 70 cycles per minute in a reciprocal shaking water bath. After 24-hour exposure to tobramycin, the biofilm-coated disks were removed from the culture vial, dipped in sterile PBS to remove antibiotics and unattached cells, then placed into a scintillation vial containing 5 mL sterile PBS, and enumerated using the sonication and dilution plating protocol described in Section 2.3. Following dilution plating, cells were cultured on LB to determine the total number of cells and LB plus tobramycin (2 µg.mL^−1^ for *E.* coli and 0.5 µg.mL^−1^ for *P. aeruginosa*) to determine resistance.

### 2.6. Antibiotic and Bacteriophage Treatment

Forty-eight-hour biofilms were gently rinsed with 5 mL of PBS in sterile test tubes to remove planktonic cells. Biofilms were placed in individual 20 mL scintillation vials containing 10 mL of LB broth with tobramycin (2 µg.mL^−1^) and T4 (MOI of 0.01) for *E.* coli and tobramycin (0.5 µg.mL^−1^) and PB-1 (MOI of 0.01) for *P. aeruginosa*. Biofilms were incubated 70 cycles per minute in a reciprocal shaking water bath at 37 °C. After 24 h biofilm growth was measured using the sonication and dilution plating protocol described in Section 2.3. Tobramycin resistant cells were determined as described in Section 2.5. Phage resistant cells were determined by plating bacterial dilutions onto LB agar followed by an agar overlay containing 0.1 mL of 10^8^ PFU.mL^−1^ and measuring bacterial colonies following 24-hour incubation.

### 2.7. Data Analysis

A minimum of three biological replicates were performed for each experiment. CFU data was log transformed to ensure a normal distribution. When bacterial cell counts were less than the detection limit (<20 CFU.mL^−1^), the experiment was repeated, and, if the result was confirmed, the CFU data was recorded as 1 (log_10_ (1) = 0). Data was analyzed by ANOVA using Sigma Plot v 12.5 [27] and Holm-Sidak *post hoc* tests were used to compare the effects of antibiotic and phage treatment results against untreated controls.

## 3. Results and Discussion

### 3.1. Effect of Antibiotic and Phage Concentrations on Cell Survival

*E. coli* and *P. aeruginosa* 48 h biofilms were treated with varying concentrations of tobramycin (0.25–4 µg.mL^−1^) and phage concentrations (from 10^−4^ to 10^1^ multiplicity of infection (MOI)) for 24 h followed by viable cell assays to determine cell survival. During preliminary experiments [28], antibiotic and phage concentrations were chosen so that individual treatments resulted in a ten-fold reduction (*i.e*., 90% kill) in CFU, and potential phage-antibiotic interactions could be assessed. As a result of preliminary tests, antibiotic concentrations of 2 µg.mL^−1^ and 0.5 µg.mL^−1^ were used to treat *E. coli* and *P. aeruginosa* biofilms respectively. Similarly, a MOI of 0.01 was used for phage treatment of *E. coli* biofilms (T4 phage) and *P. aeruginosa* biofilms (PB-1 phage).

### 3.2. Effect of Tobramycin and T4 on *E. coli* Biofilm Cell Survival

**Figure 1:**
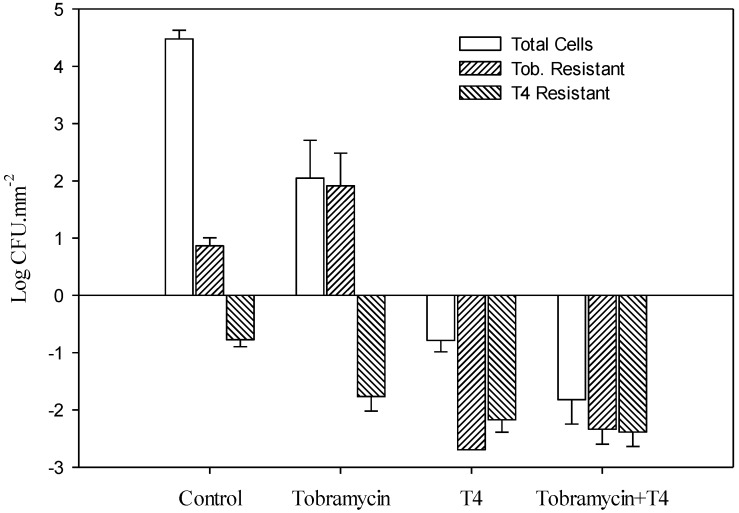
Effect of 24-hour exposure of preformed 48-hour-old *E. coli* biofilms to tobramycin, T4, and the combination of tobramycin and T4 phage on total cell concentrations and concentrations of tobramycin and phage (T4) resistant populations. The differences of bacterial populations (total cells), as well as tobramycin-resistant and phage-resistant populations, were all significantly different (*p* < 0.05) from their respective untreated (control) values. Bars indicate standard error of the mean.

*E. coli* biofilms were exposed to tobramycin or a combination of tobramycin and T4 for 24 h followed by viable cell counts to determine the number of surviving cells (Table 1 and Figure 1). Tobramycin or the combination resulted in 2.1 ± 0.66 and −1.8 ± 0.43 CFU.mm^–2^ (log mean ± SE) of the biofilm remaining, respectively. The combination of phage and antibiotic led to ~99.99% decrease on the survival of *E. coli* biofilms compared to the use of tobramycin alone. Similar results have been found in studies on *Klebsiella pneumoniae* and *Staphylococcus aureus* biofilms using an antibiotic combined with phage [20,21,22].

**Table 1:** Survival of *E. coli* and *P. aeruginosa* biofilm cells treated with bacteriophage and tobramycin. Untreated (control) values are also listed.

	*E. coli*			*P. aeruginosa*		
Time (h)	Log CFU.mm^–2^ ± SE					
6 (Control)	4.39 ± 0.16			2.96 ± 0.15		
24 (Control)	4.48 ± 0.15			3.63 ± 0.15		
Treatment	Tobramycin ^a^	T4 ^a^	Tob + T4 ^a^	Tobramycin ^b^	PB-1 ^b^	Tob + PB-1 ^b^
6	−0.27 ± 0.55	−0.10 ± 0.21	−1.6 ± 0.32	2.7 ± 0.30	2.4 ± 0.21	2.0 ± 0.35
24	2.1 ± 0.66	−0.79 ± 0.20	−1.8 ± 0.43	1.8 ± 0.36	3.2 ± 0.17	1.6 ± 0.33

^a^: log Mean ± SE (*n* = 12); ^b^: log Mean ± SE (*n* = 15).

### 3.3. Determination of E. coli Biofilm Cell Resistance

Following 24 h exposure to tobramycin, T4, or their combination, biofilms were assayed to determine tobramycin resistant cells and T4 resistant cells (Table 2 and Figure 1). There was a >99.99% decrease observed in tobramycin resistant cells and a 39% decrease in the number of T4 resistant cells in the combination treatment.

**Table 2:** Resistance to tobramycin and bacteriophage in *E. coli* and *P. aeruginosa* biofilms.

	*E. coli*		*P. aeruginosa*	
% decrease in resistance	Tobramycin ^a^	T4 ^b^	Tobramycin ^c^	PB-1 ^d^
	>99.99%	39%	60%	99%

^a^: % decrease in resistance following Tob + T4 treatment compared to tobramycin alone; ^b^: % decrease in resistance following Tob + T4 treatment compared to T4 alone; ^c^: % decrease in resistance following Tob + PB-1 treatment compared to tobramycin alone; ^d^: % decrease in resistance following Tob + PB-1 treatment compare to PB-1 alone.

### 3.4. Effect of Tobramycin and PB-1 on *P. aeruginosa* Biofilm Cell Survival

**Figure 2:**
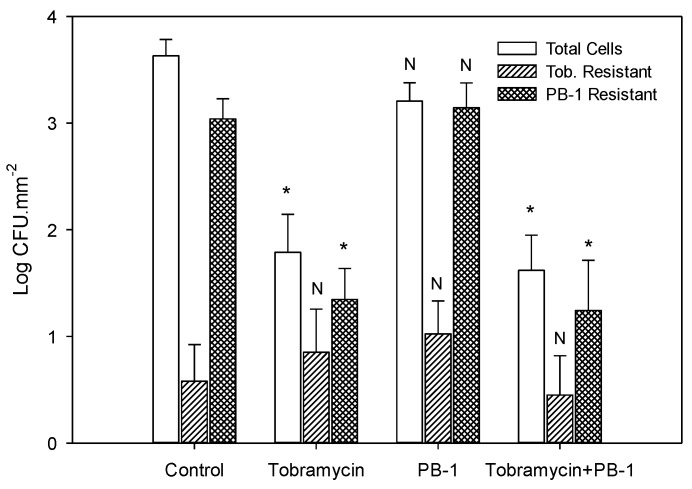
Effect of 24-hour exposure of preformed 48 h old *P. aeruginosa* biofilms to tobramycin, PB-1, and the combination of tobramycin and PB-1 on total cell concentrations and concentrations of tobramycin (Tob.) and phage (PB-1) resistant populations. Differences between untreated control values Figureand treated values are denoted by * (statistically significant *p* < 0.05) or N (not statistically significant, *p* > 0.05). Bars indicate standard error of the mean.

*P. aeruginosa* biofilms were exposed to tobramycin or a combination of tobramycin and PB-1 for 24 h and the number of surviving cells were determined (Table 1 and Figure 2). Treatment with tobramycin or the combination resulted in 1.8 ± 0.36 and 1.6 ± 0.33 CFU.mm^−2^ (log Mean ± SE) remaining, respectively. The combination of tobramycin and PB-1 on *P. aeruginosa* biofilms was just as effective as tobramycin alone in decreasing biofilm mass. The high level of resistance observed to phage PB-1 in *P. aeruginosa* biofilms may be due to EPS restricting the ability of the phage to access receptors [29].

## 3.5. Determination of *P. aeruginosa* Biofilm Cell Resistance

Following 24-hour exposure to tobramycin, PB-1, and their combination, biofilms were assayed to determine tobramycin resistant cells and PB-1 resistant cells (Table 2 and Figure 2). To determine the percent decrease in resistant cells, the number of resistant cells in the combination treatment was compared to the number of resistant cells in the tobramycin and PB-1 treatment. There was a 60% decrease in the number of tobramycin resistant cells and a 99% decrease in the number of PB-1 resistant cells in the combination treatment.

The current study found the combination of tobramycin and T4 on *E. coli* biofilms led to a >99.99% decrease in tobramycin resistant cells and a 39% decrease in T4 resistant cells compared to use the use of tobramycin or phage alone, respectively. Treating *P. aeruginosa* biofilms with the combination of tobramycin and PB-1 resulted in a 60% decrease in tobramycin resistant cells and a 99% decrease in phage resistant cells compared to the use of tobramycin or phage alone, respectively. These results agree with other studies which showed that combinations of either antibiotics alone or phage alone can result in a decrease in the emergence of resistant cells [16,17,19].

As stated earlier, there is considerable work showing how phage can move through bacterial EPS components of biofilm matrices, and attach to cell surface targets by virtue of their production of depolymerase enzymes [21,29,30]. However, most of these studies are associated with pure bacterial cultures. One issue that needs to be addressed in future work is the efficacy of combination antibiotic-phage therapy in treating polymicrobial biofilm infections. Polymicrobial infections including those associated with wounds [31,32] are often much more recalcitrant to antibiotic treatment than are monoculture infections. In a recent study, we observed that a phage-susceptible bacterial species can be protected within a mixed culture biofilm by virtue of the phage-host bacterium association with a different species that would produce a chemically-different EPS, not susceptible to the highly specific phage depolymerase [33]. In this context, future studies on combination therapy involving bacteriophage should explore the effectiveness against mixed cultures.
